# 

**Published:** 1915-11

**Authors:** 


### CONTENTS, NOVEMBER, 1915—VOLUME XXXI, NO. 5.

ORIGINAL ARTICLES.

President's Address—The Texas Surgical Society. By James E. Thompson, M. D., Galveston, Texas ........ 177

The Thyroid Gland. By W. L. Crosthwait, M. D., Waco, Texas ........ 183

DEPARTMENT OF EUGENICS.

Alcohol, Syphilis, and Gonorrhea, on Death Certificates ........ 191

Genetic Sentilations ........ 192

To the Rescue ........ 195

The Moral Transition ........ 197

EDITORIAL DEPARTMENT.

Fads in Medicine ........ 200

Meeting of Southern Medical Association ........ 201

Medical History of Texas ........ 204

Two Great Men Gone ........ 205

Dr. Oscar Dowling, President of the Southern Medical Association ........ 206

Items of General Interest ........ 208

Personal Mention ........ 215

Personals ........ 217

Sanitariums ........ 218

Marriages ........ 218

Deaths ........ 220

Helpful Hints for the Busy Doctor ........ 220

